# Supplementary material for: Pore-pressure diffusion controls upper-plate aftershocks of the 2014 Iquique earthquake
Source: Nat Commun. 2025 Oct 27;16:9474. doi: 10.1038/s41467-025-65013-6 (PMC12559721; doi:10.1038/s41467-025-65013-6)
Supplement: Supplementary file 1 — Supplementary Information [file 41467_2025_65013_MOESM1_ESM.pdf]

Supplementary Information for:

Pore-pressure diffusion controls upper-plate aftershocks of the 2014 Iquique earthquake

Carlos Peña[1,2*], Oliver Heidbach[2,3], Sabrina Metzger[2], Bernd Schurr[2], Marcos Moreno[4], Jonathan Bedford[5], Onno Oncken[2,6], and Claudio Faccenna[2,7]

[1]Institute of Geosciences, University of Potsdam, Potsdam, Germany
[2]GFZ Helmholtz Centre for Geosciences, Potsdam, Germany
[3]Technical University of Berlin, Berlin, Germany
[4]Department of Structural and Geotechnical Engineering, Pontificia Universidad Católica de Chile, Santiago, Chile
[5]Institute of Geosciences, Ruhr-University Bochum, Bochum, Germany
[6]Department of Earth Sciences, Free University Berlin, Berlin, Germany
[7]Dipartimento di Scienze, Università Roma Tre, Rome, Italy
[*]Corresponding author, carlosp@gfz.de

Supplementary Figures 1–29

Figure 1. Magnitude of the seismicity events.
Figure 2. GNSS Time-series fits.
Figure 3. Individual postseismic surface displacements.
Figure 4. Mean absolute errors.
Table 1. F-test.
Figure 5. Shear and normal stresses along a cross-section at 12.5 km depth.
Figure 6. Shear and normal stresses along the profile P–P'.
Figure 7. Poroelastic and elastic-only ΔCFS values along the profile P–P'.
Figure 8. Additional cross-section profiles showing pore-pressure changes and upper-plate aftershocks.
Figure 9. Aftershock migration front a discrete source-point fluid model.
Figure 10. 3D model geometry and element size.
Figure 11. Second-order element resolution test.
Figure 12. Impact of K_f and v_u on pore-pressure changes along a cross-section at 12.5 km depth.
Figure 13. As Fig. 12 along the profile P–P'.
Figure 14. Observed versus predicted coseismic displacements.
Figure 15. Difference between coseismic deformation models in percentage.
Figure 16. Effect of poroelasticity and viscoelasticity on afterslip inversions.
Figure 17. Afterslip model resolution.
Figure 18. Coupled versus uncoupled model test.
Figure 19. Equivalent strain calculation.
Figure 20. Second invariant of the deviatoric stress tensor calculation.
Figure 21. Impact of friction coefficient on ΔCFS from afterslip at a cross-section at 12.5 km depth.
Figure 22. As Fig. 21 for the profile P–P'.
Figure 23. As Fig. 21 but considering poroelasticity.
Figure 24. As Fig. 22. for the profile P–P'.
Figure 25. ΔCFS produced by afterslip considering an elastic-only model inversion along a cross-section at 12.5 km depth.
Figure 26. ΔCFS produced by afterslip considering an elastic-only model inversion along the profile P–P'.
Figure 27. Comparison of the surface displacements produced by the main shock and the largest aftershock M_w = 7.6.
Figure 28. Coseismic ΔCFS along a cross-section at 12.5 km depth.
Figure 29. Coseismic ΔCFS along the profile P–P'.

Supplementary Figures:

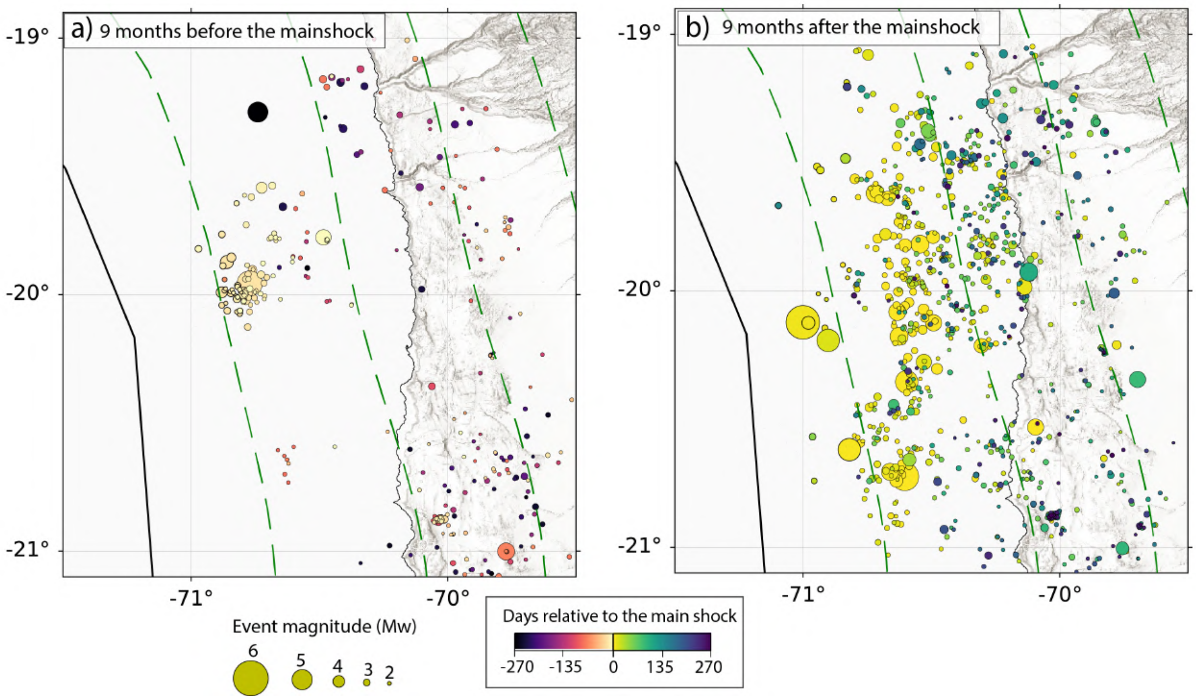

Figure 1. **Magnitude of the seismicity events**. Seismicity before (a) and after the main shock (b), color-coded by time and scaled by event-size.

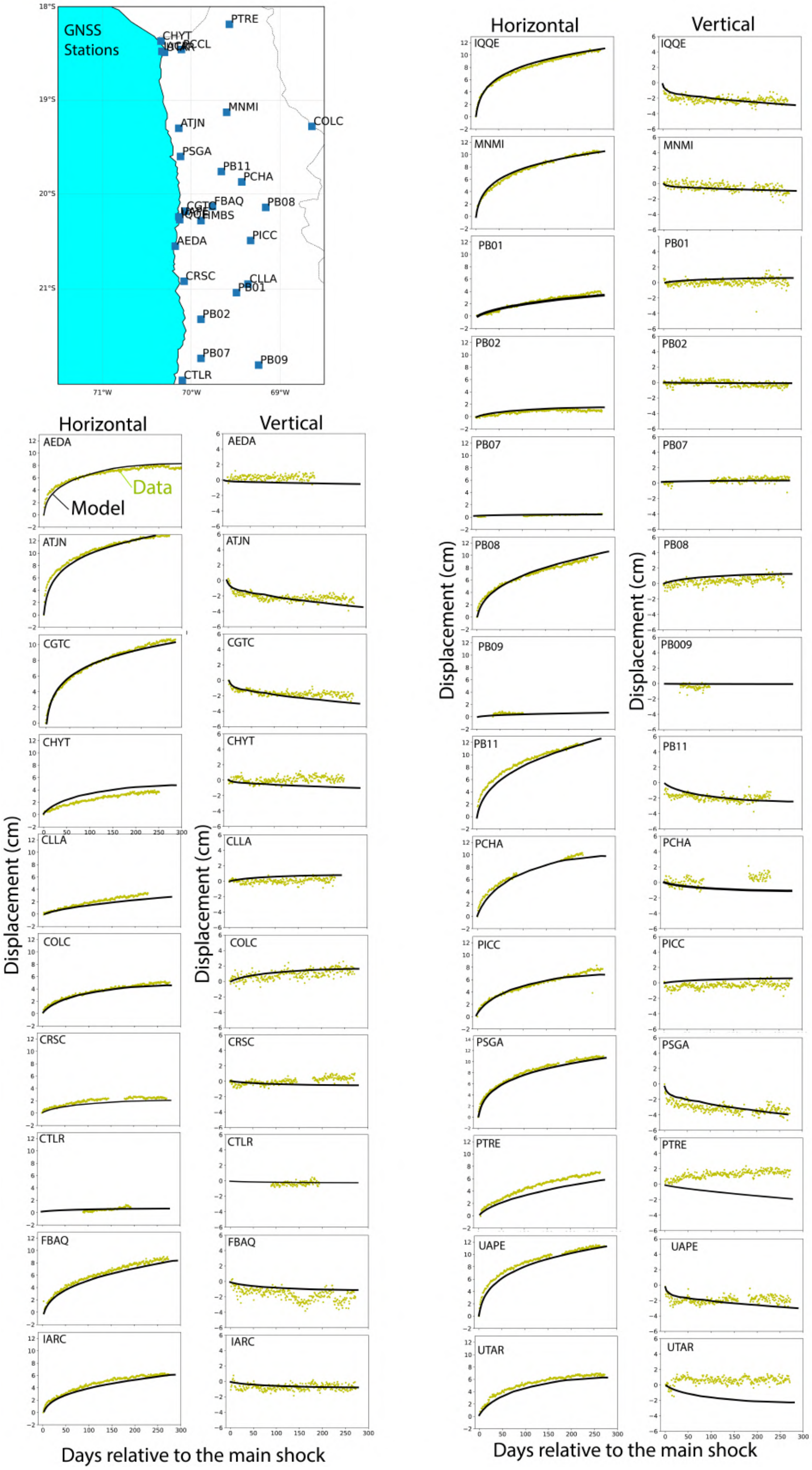

Figure 2. **Geodetic Time-series fits**. Observed and modelled Global Navigation Satellite System (GNSS) time series displacements.

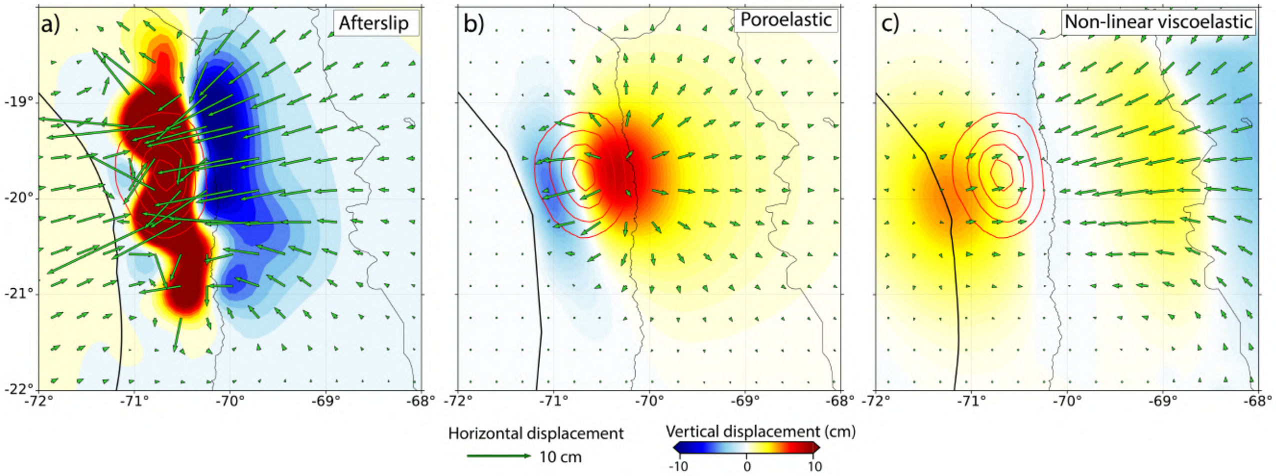

Figure 3. Individual postseismic surface displacements. Cumulative surface displacement field after 270 days due to afterslip (a), poroelasticity (b), and non-linear viscoelastic relaxation (c).

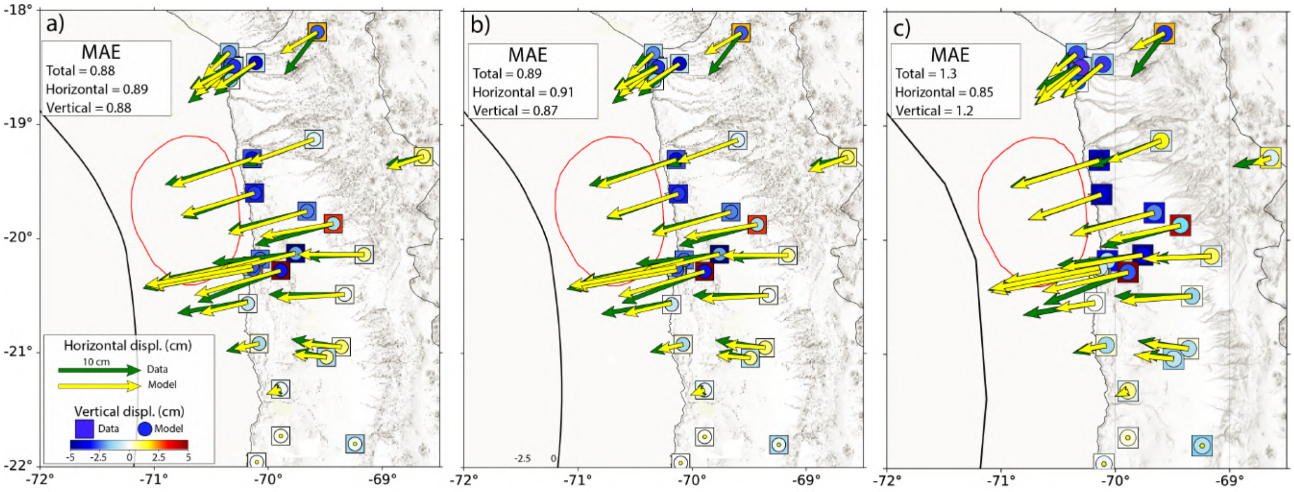

Figure 4. Mean absolute errors. Mean absolute error (MAE) for the observed and predicted accumulated postseismic geodetic displacements. Panel a depicts the total fit, incorporating viscoelastic, poroelastic, and afterslip processes (total MAE = 0.88), panel b shows the fit considering only viscoelastic and afterslip processes (total MAE = 0.89), and panel c presents the model fit considering only afterslip (total MAE = 1.3).

Test	Simulation	Null hypothesis simulation	(F-test) p-value
1)	AF + PE + VER	AF	1×10^{-14}
2)	AF + PE + VER	AF + VER	1×10^{-5}

Table 1. Statistical F-test results. We performed two F-tests by comparing the resulting surface displacements from preferred model considering all postseismic processes, i.e., afterslip (AF), poroelastic (PE), and viscoelastic relaxation (VER) processes to the surface displacements resulting from 1) afterslip only and 2) afterslip and viscoelastic relaxation simulations. The F-tests follow the approach described in Peña et al.[1]. Here, the two null hypotheses are: 1) a simulation considering afterslip only provides a significant better fit to the geodetic data than a simulation considering all three (AF+PE+VER) postseismic deformation processes, and 2) a simulation considering afterslip and viscoelastic relaxation produces a significant better fit than a simulation considering all three postseismic deformation processes. The latter is particularly useful to evaluate if the inclusion of poroelasticity in a simulation considering afterslip and viscoelastic relaxation is statistically significant and better than excluding poroelasticity. We consider a standard significance value of 0.05[2]. For the 1) and 2) tests, we find statistical p-values using the F-test of approximately 10^{-14} and 10^{-5}, respectively. These values align well with findings from studies employing highly dense geodetic measurements[1,2] and are significantly smaller than the standard significance level of 0.05, leading to the rejection of the null hypotheses, i.e., including poro- and viscoelasticity to a model considering afterslip substantially increases its statistical significance. In particular, our results from the test 2) indicate that including poroelasticity is more than three orders of magnitude more significant than excluding it.

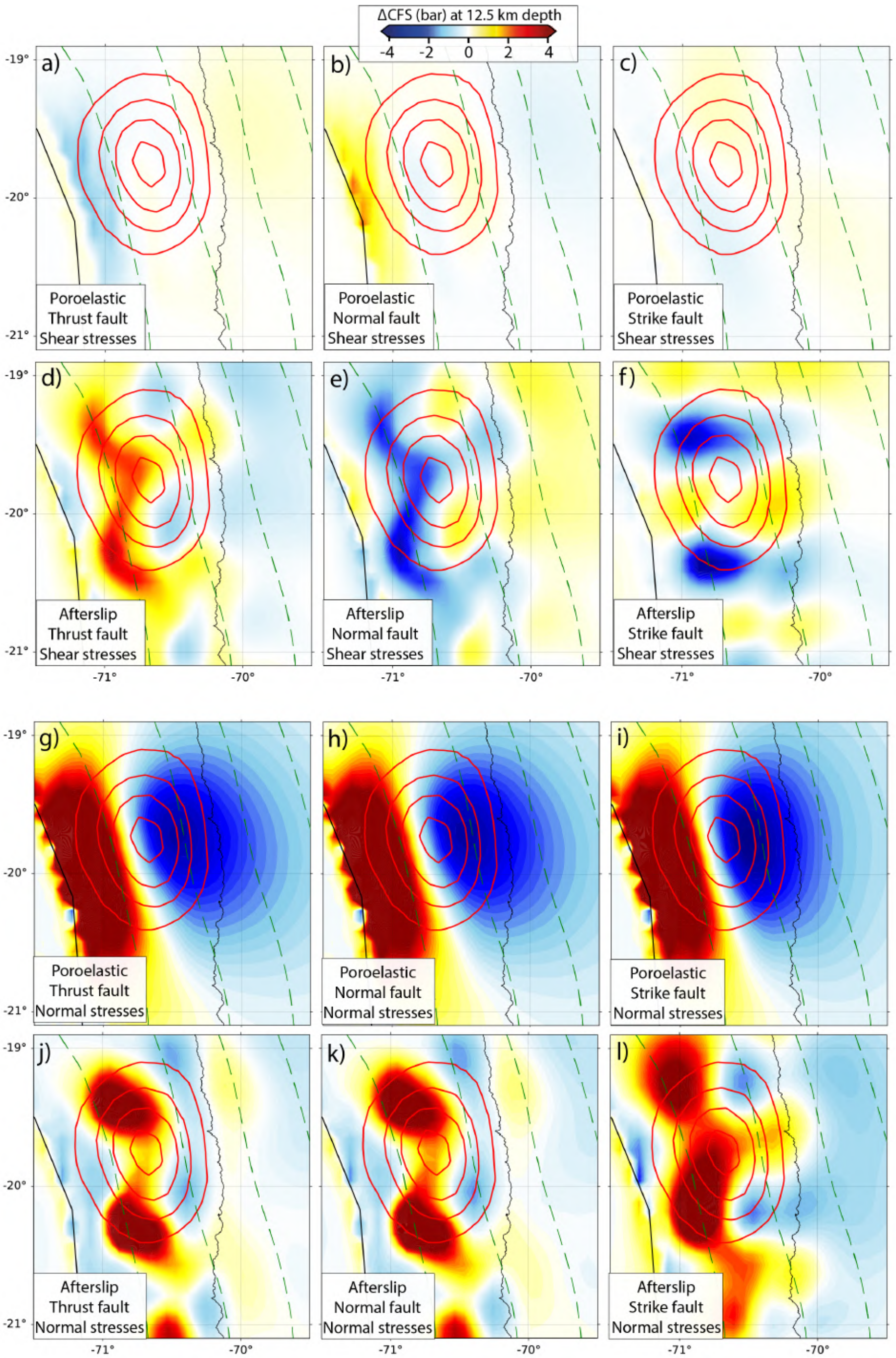

Figure 5. Shear and normal stresses along a cross-section at 12.5 km depth. Shear and normal stress components of the Coulomb Failure Stress changes (ΔCFS) shown in Fig. 3 (main text), resulting from poroelastic and afterslip processes. Shear stresses due to poroelastic and afterslip processes are presented in the first (a–c) and second (d–f) rows, respectively. Normal stresses from poroelastic and afterslip processes are shown in the third (g–i) and fourth (j–l) rows, respectively. The first (a, d, g, j), second (b, e, h, k), and third (c, f, i, l) columns illustrate the stresses for thrust, normal, and strike-slip faults, respectively. Further details about the receiver fault geometry can be found in Fig. 3 (main text).

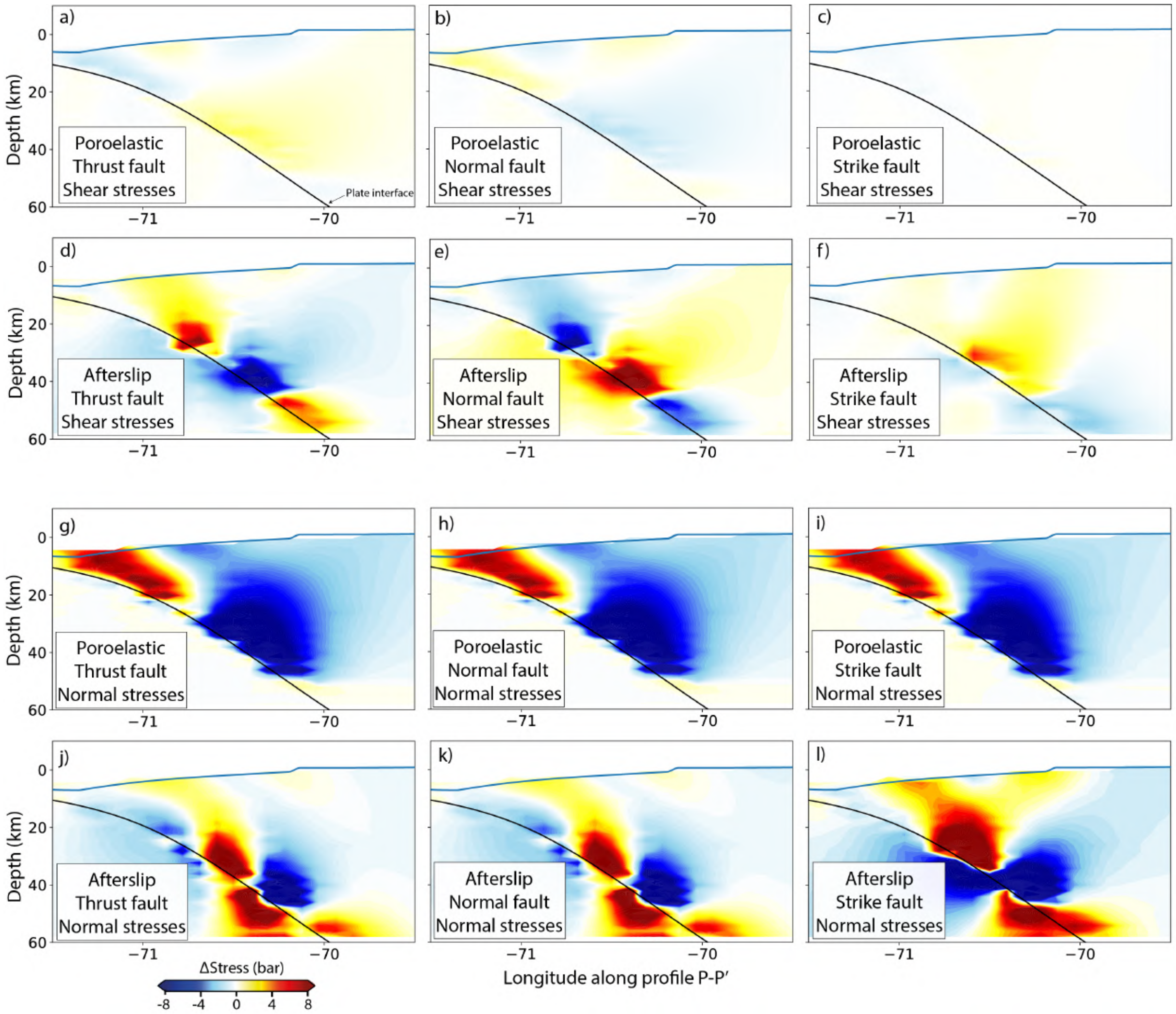

Figure 6. Shear and normal stresses along the profile P–P'.. As shown in Fig. 5, along a W-E cross section P-P' at 19.75°S.

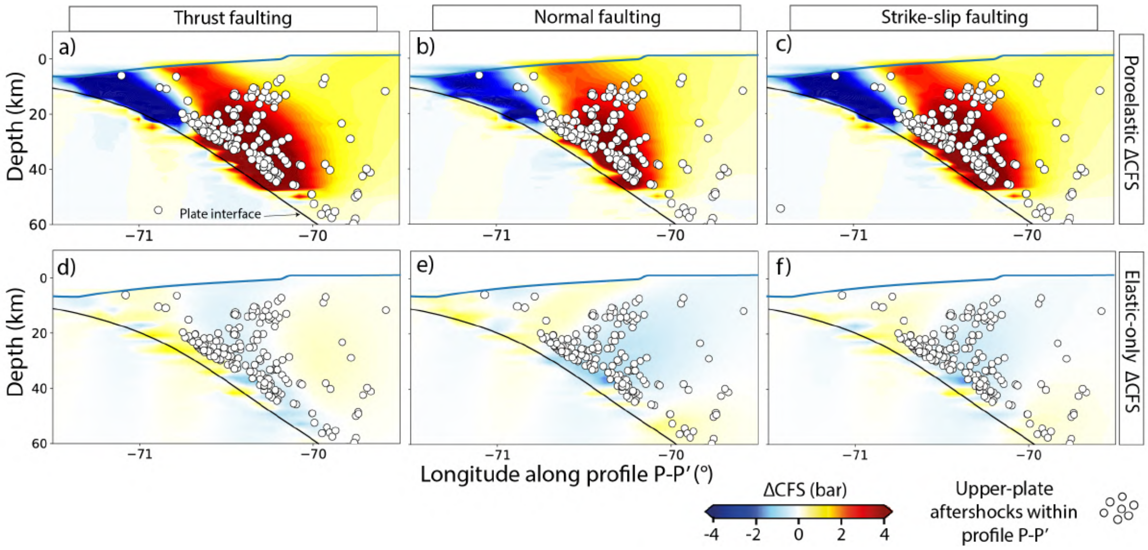

Figure 7. **Poroelastic and elastic-only Coulomb Failure stress changes values along the profile P–P’**. Cumulative Coulomb Failure Stress Changes (ΔCFS) after 270 days due to poroelastic stresses (a–c) compared to those resulting from elastic-only stresses (d–f). Note that most of the ΔCFS are driven by pore-pressure changes.

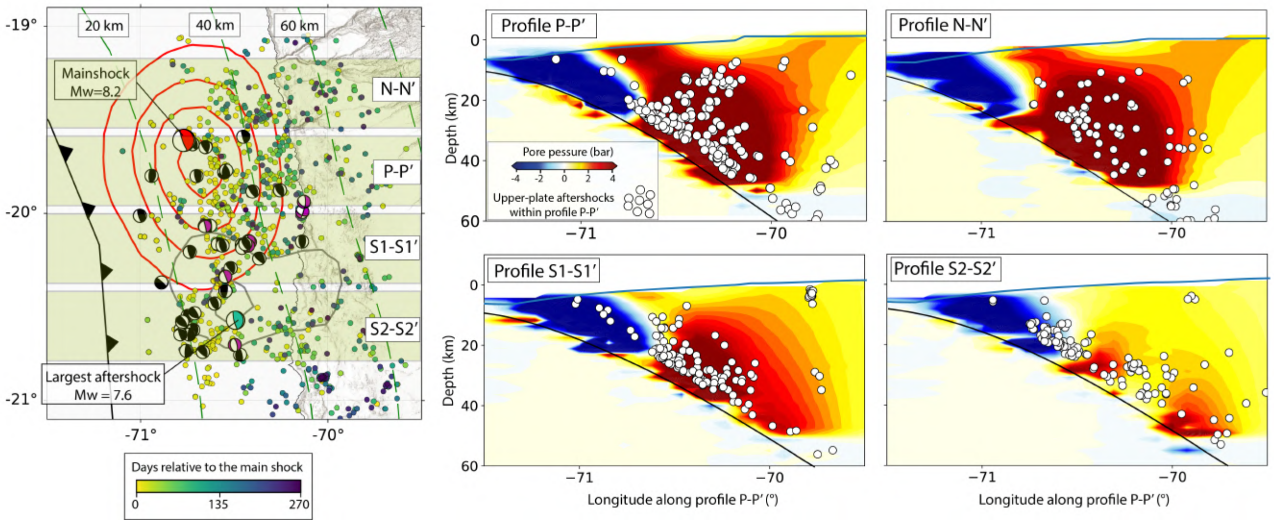

Figure 8. **Additional cross-section profiles showing pore-pressure changes and upper-plate aftershocks**. Cumulative distribution of postseismic pore-pressure changes after 270 days along different profiles in the northern (N–N’), center (P–P’), southern (S1–S1’) segments, as well as in the region of the largest aftershock (S2–S2’).

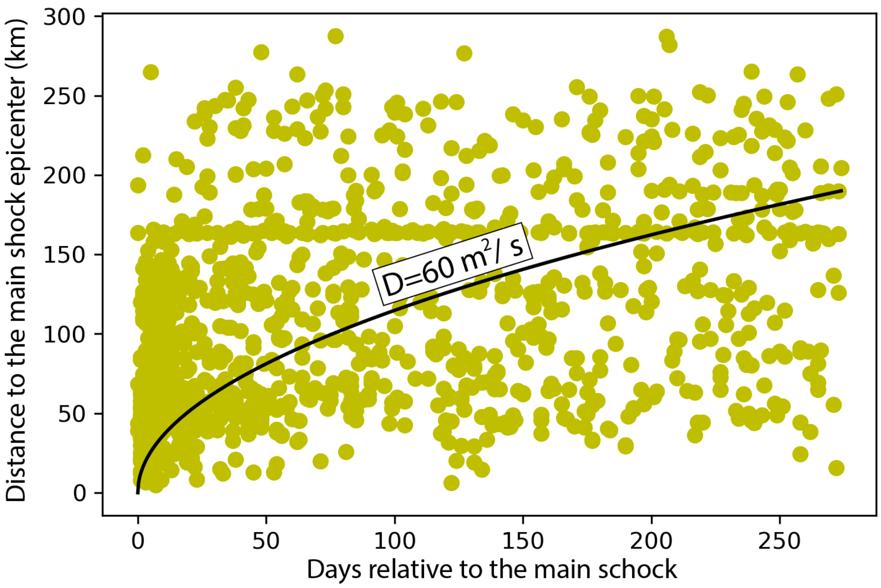

Figure 9. Aftershock migration front a discrete source-point fluid model. Diffusivity (D) obtained following Shapiro et al.[3]. The model parameters to compute permeability from diffusivity are the ones stated in the Method section in the main text.

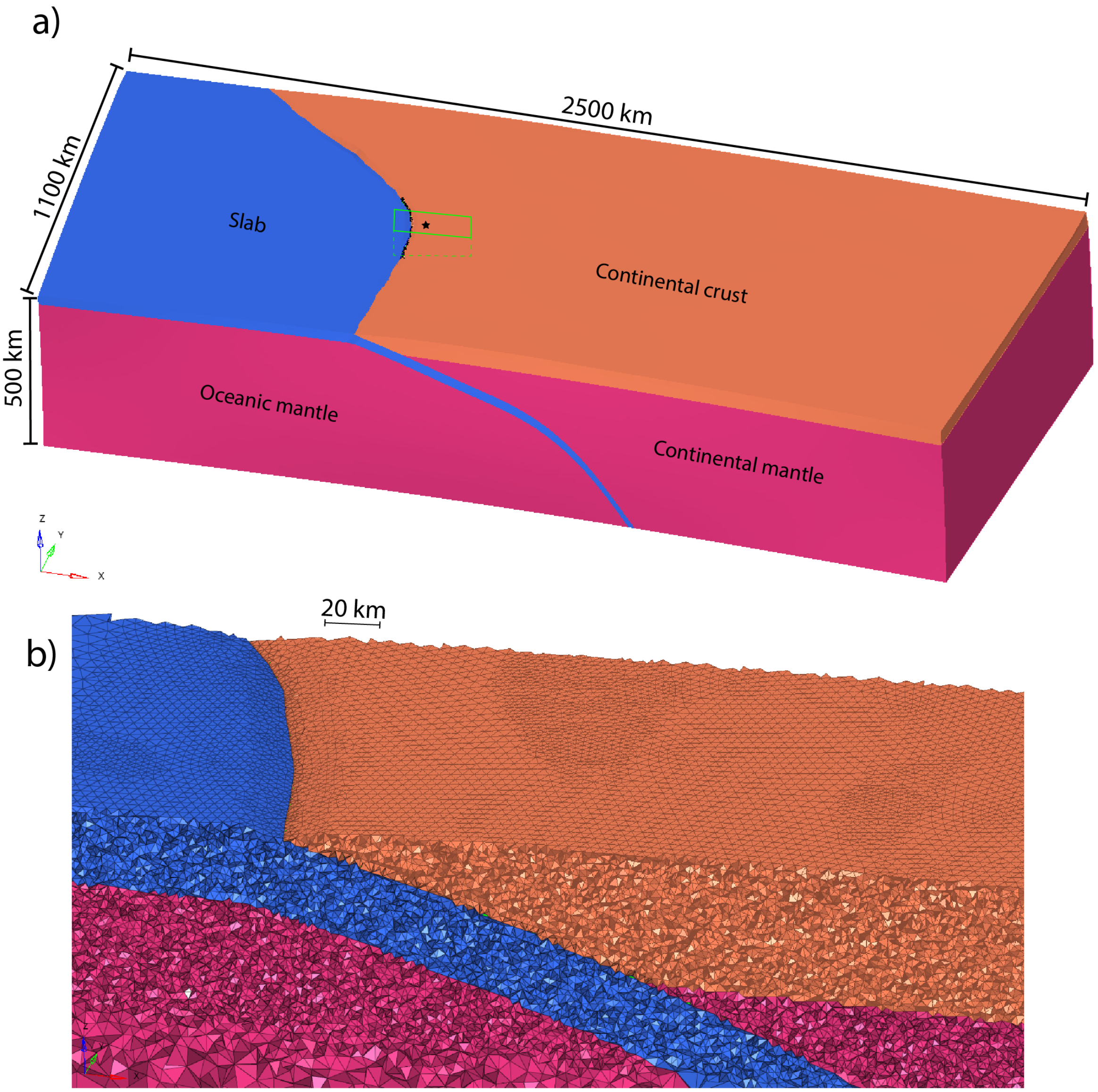

Figure 10. Model geometry and element size. Model geometry (a) and a subsection of the model showing the element size and discretization in the region of key postseismic deformation (b), obtained from the yellow polygon displayed in a). Black star in a) illustrates the epicenter of the main shock.

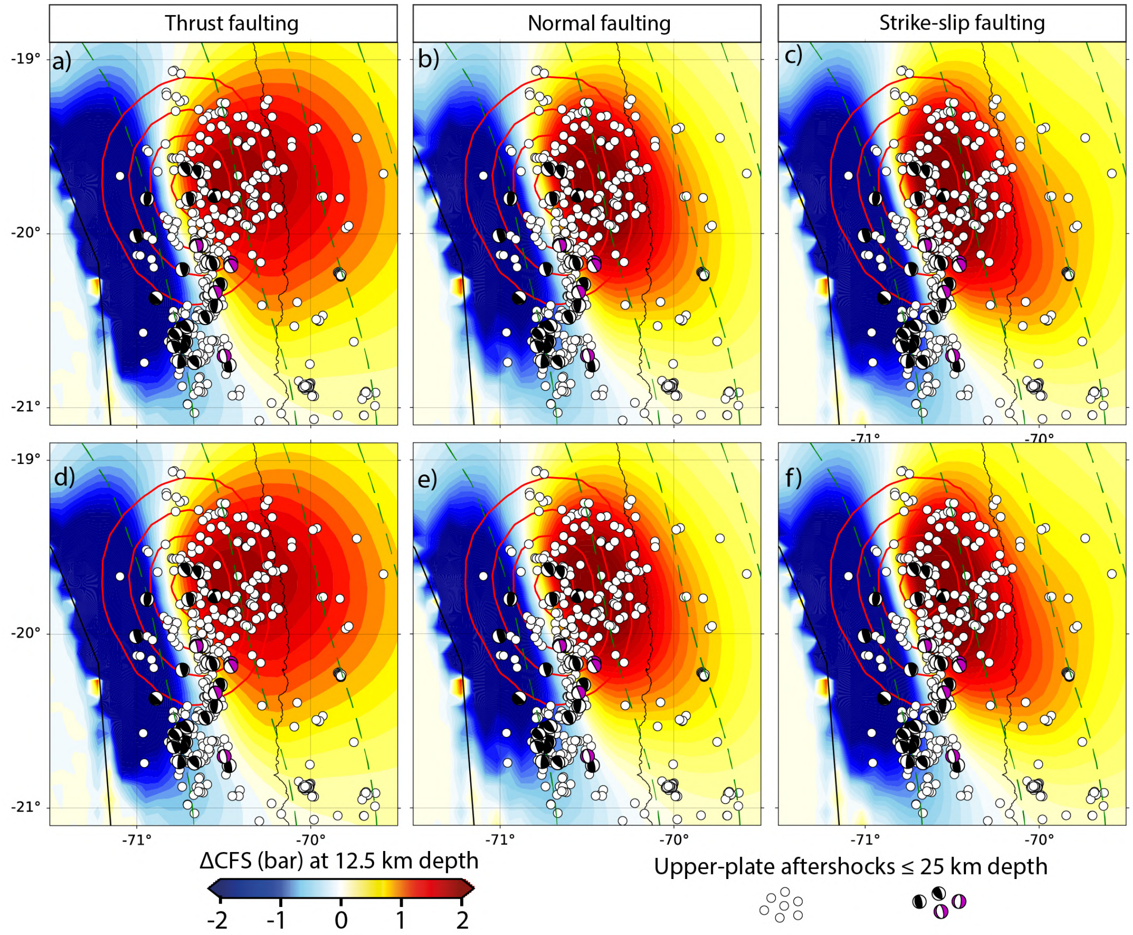

Figure 11. **Second-order element resolution test**. Second-order element resolution test. The upper panel (a–c) shows the resulting ΔCFS at a depth of 12.5 km, as presented in Fig. 3 of the main text. The lower panel (d–f) illustrates the results obtained using second-order elements, which incorporate additional mid-side nodes within the elements in the region of key postseismic deformation, for comparison with the upper panel.

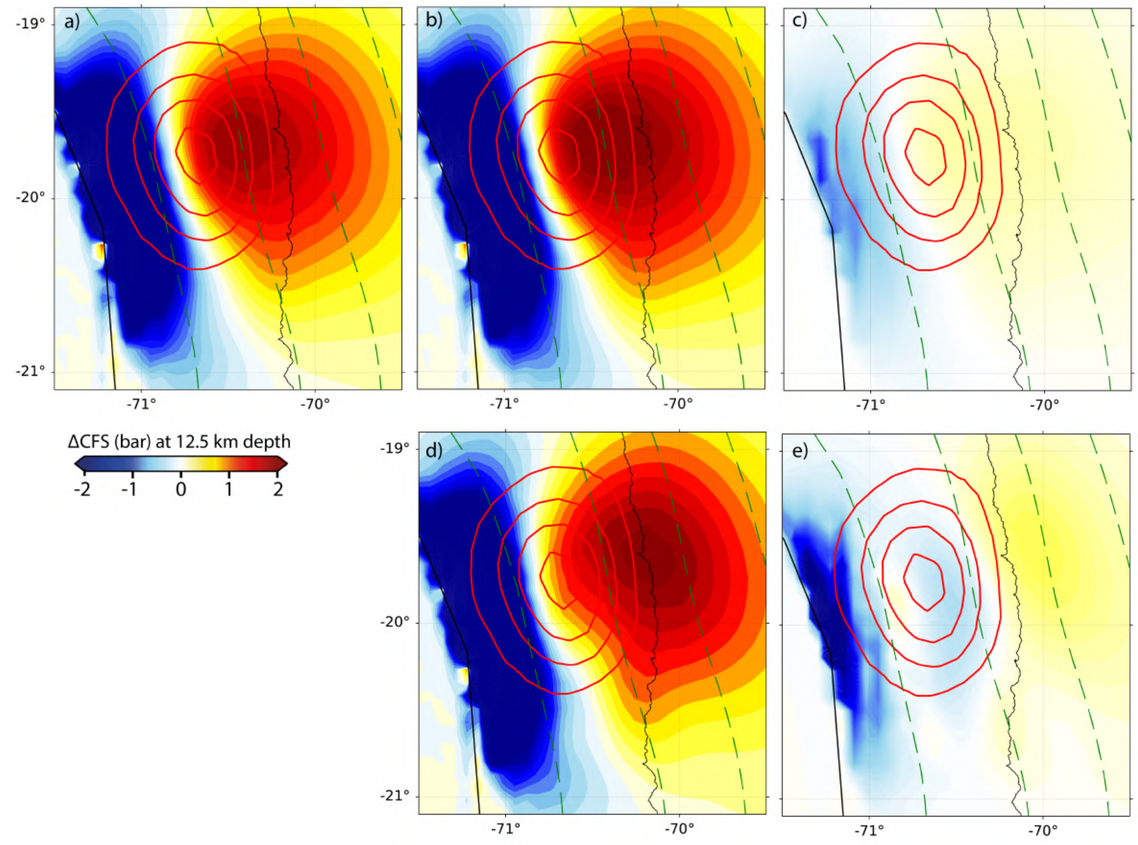

Figure 12. **Impact of pore-fluid (water) bulk K_f and undrained poisson's ratio ν_u on the postseismic Coulomb Failure Stress changes (ΔCFS) for a thrust receiver fault resulting from poroelasticity at a depth of 12.5 km**. a) ΔCFS from our model with K_f = 2.8 GPa and and ν_u = 0.34. b) ΔCFS from our model with and end member value of K_f = 4.0 GPa and and ν_u = 0.34. c) Difference between results of a) and b). d) ΔCFS from our model with K_f = 2.8 GPa and ν_u = 0.40. e) Difference between results of a) and d).

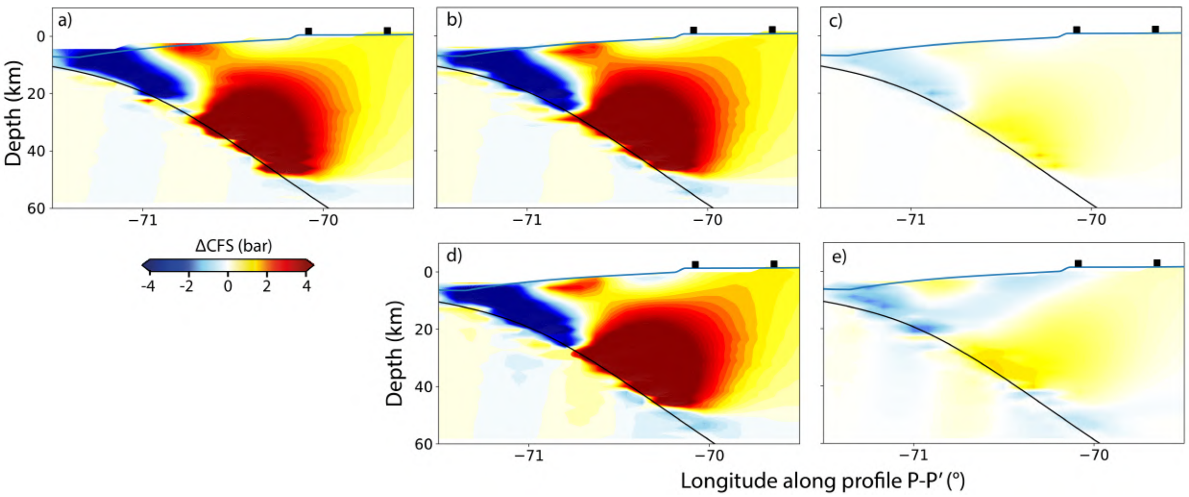

Figure 13. **Impact of pore-fluid (water) bulk K_f and undrained poisson's ratio ν_u on the postseismic Coulomb Failure Stress changes (ΔCFS) for a thrust receiver fault resulting from poroelasticity along section section P–P' at 19.75°S**. The black rectangles on the surface of the model (solid blue line) indicate the locations of the two GNSS stations, PSGA (closest to the trench) and PB11, along cross section P–P'.

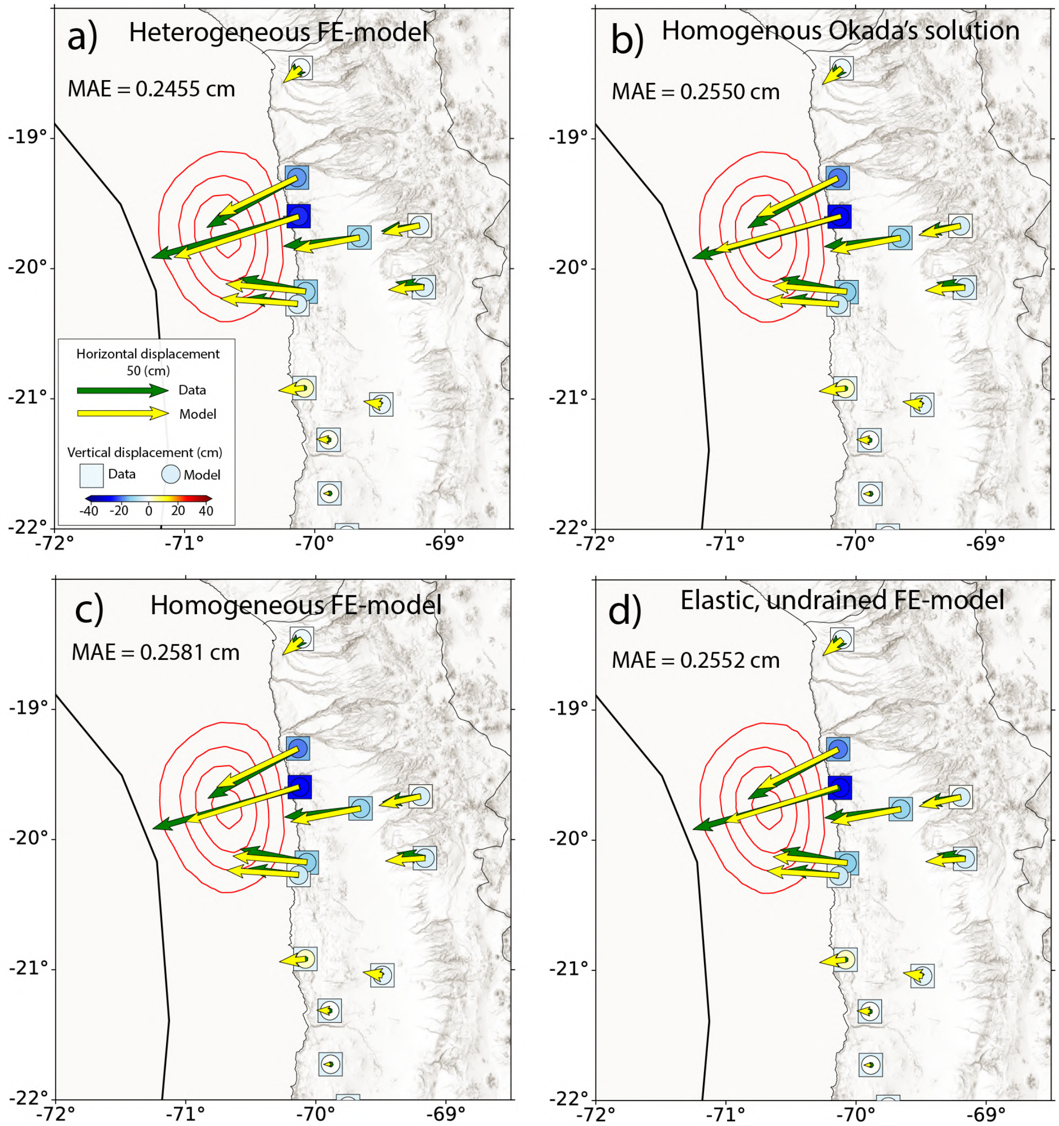

Figure 14. Observed versus predicted coseismic displacements. Coseismic geodetic observations[4] compared to displacement predictions from our finite-element model considering different rock material properties for the upper plate, slab, and upper mantle (a, Heterogeneous FE-model) (see Methods section), the half-space analytical Okada's solution[5] considering homogeneous elastic rock material properties (b), our model considering homogeneous elastic rock material in the whole domain (c), and d) as a) but considering elastic-only, undrained conditions (ν_u Poisson's ration of 0.34[6]) (d). Mean Absolute Error (MAE) is computed for each coseismic deformation simulation.

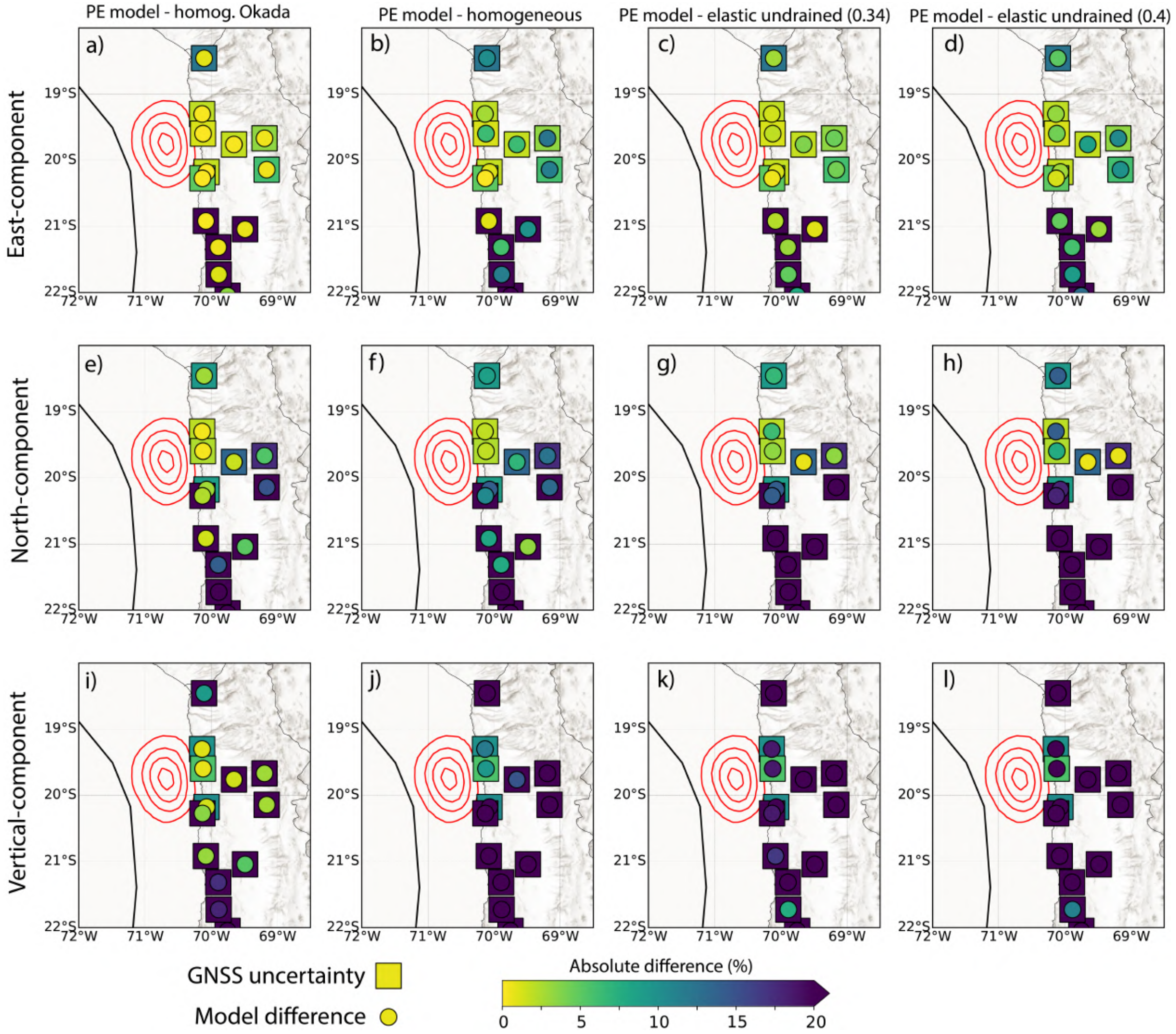

Figure 15. Difference between coseismic deformation models in percentage. Comparison of the GNSS data uncertainty with the difference between our co-seismic model (PE model) and three simulations using different rock property values displayed in Fig. 14b–d. In addition, we have included another model with ν_u=0.4. The comparison is in percentage and the color-coded squares show the GNSS data uncertainty and the circles with the same color code the difference between the models. The three rows show the three components of the deformation signal. The upper row is the difference in the W-E component, the middle row the difference in the N-S component, and the lower row the difference in the vertical component.

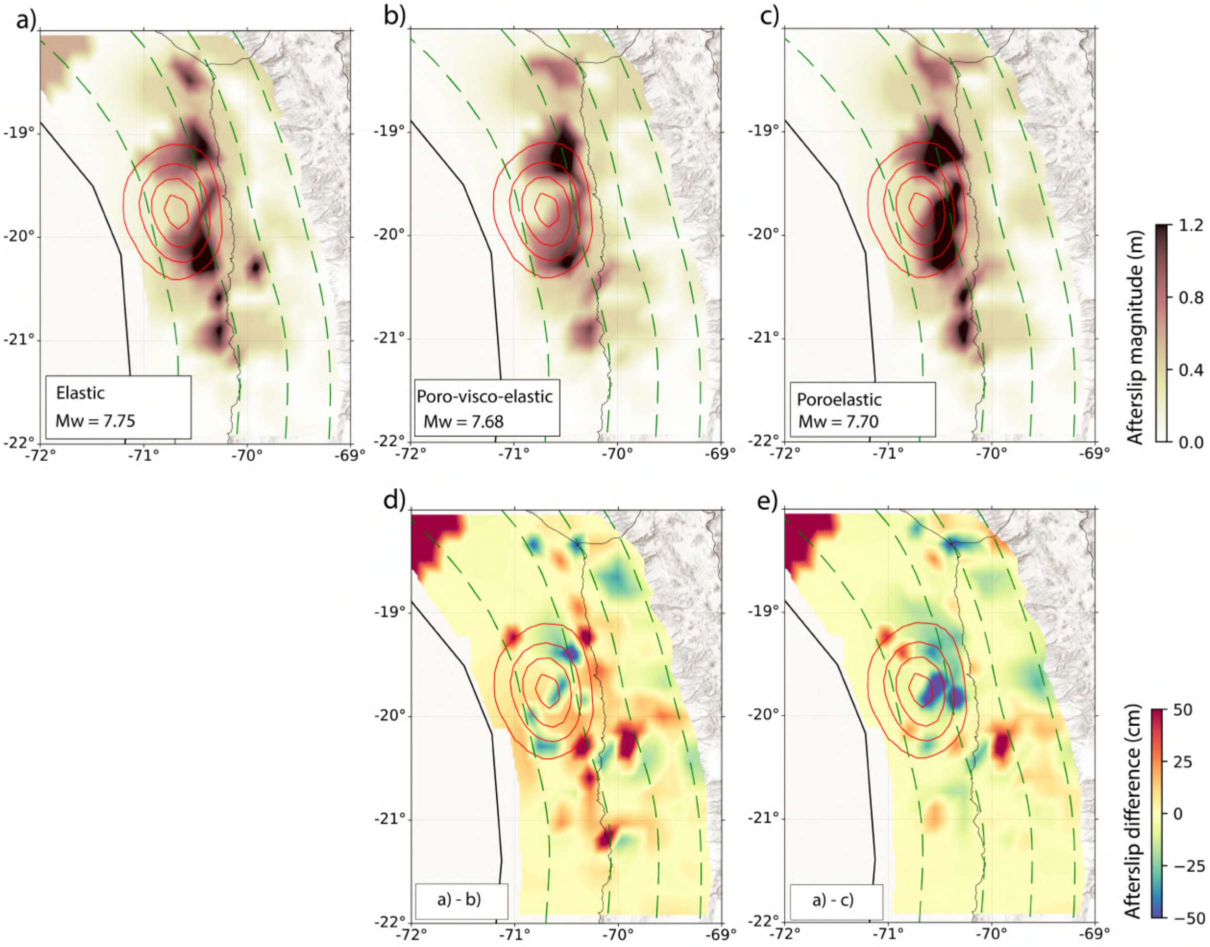

Figure 16. Effect of poroelasticity and viscoelasticity on afterslip inversions. Impact of poroelasticity and viscoelasticity on the distribution of afterslip inversions. Cumulative distribution after 270 days of afterslip using an elastic-only model (a), a poroelastic and nonlinear viscoelastic model (b), and a poroelastic-only model (c). The differences between the distributions are shown in panels (d) and (e), representing the differences between (a) and (b), and (a) and (c), respectively. Our results agree with those obtained in southern Chile[1] and Costa Rica[6].

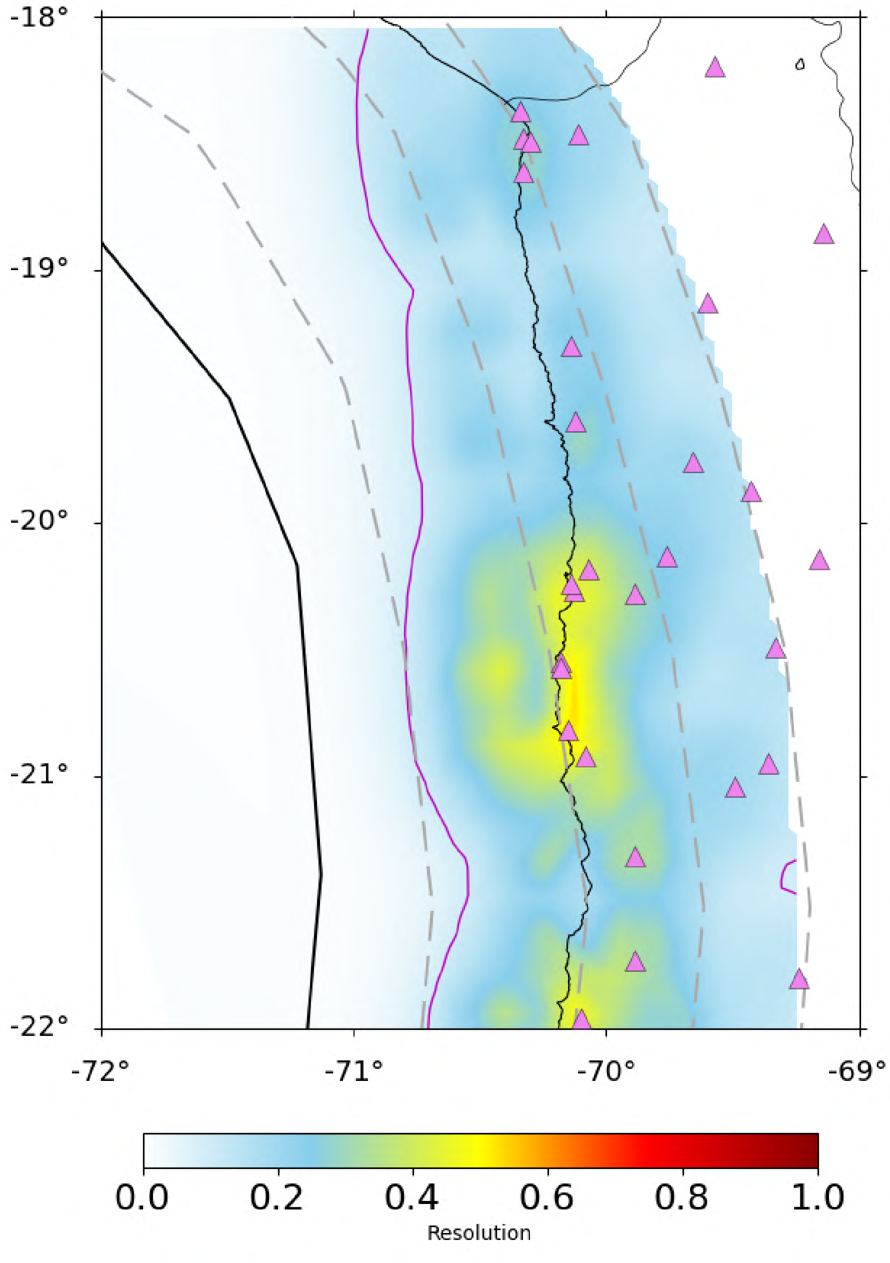

Figure 17. Afterslip model resolution. Slip resolution based on the GNSS network (pink triangles) for northern Chile, as described in Pena et al.[1]. Solid-magenta contour line represents a critical value of 0.1.

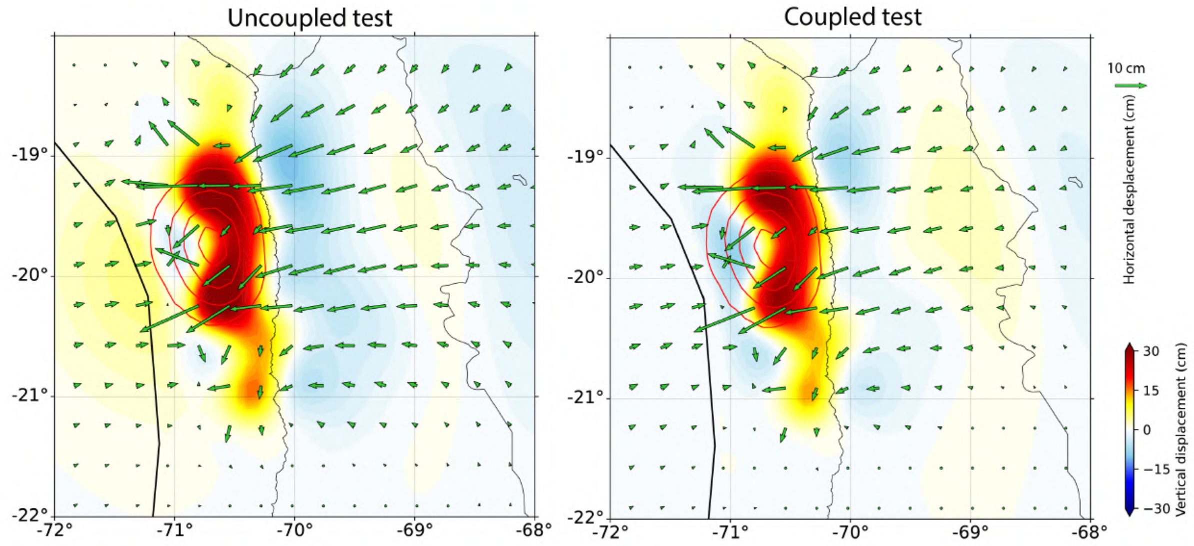

Figure 18. Coupled versus uncoupled model test. Impact of simulating postseismic deformation processes separately (a, uncoupled) or jointly (b, coupled). Following Peña et al.[1], we first run forward simulations that consider each postseismic process separately and calculate the resulting cumulative total surface displacement field after 270 days. Subsequently, we run a forward simulation that considers these three processes jointly and calculate the total surface displacement field. Consistent with our previous results in central Chile[1,7], we find no significant differences, i.e., differences much smaller than the data uncertainty.

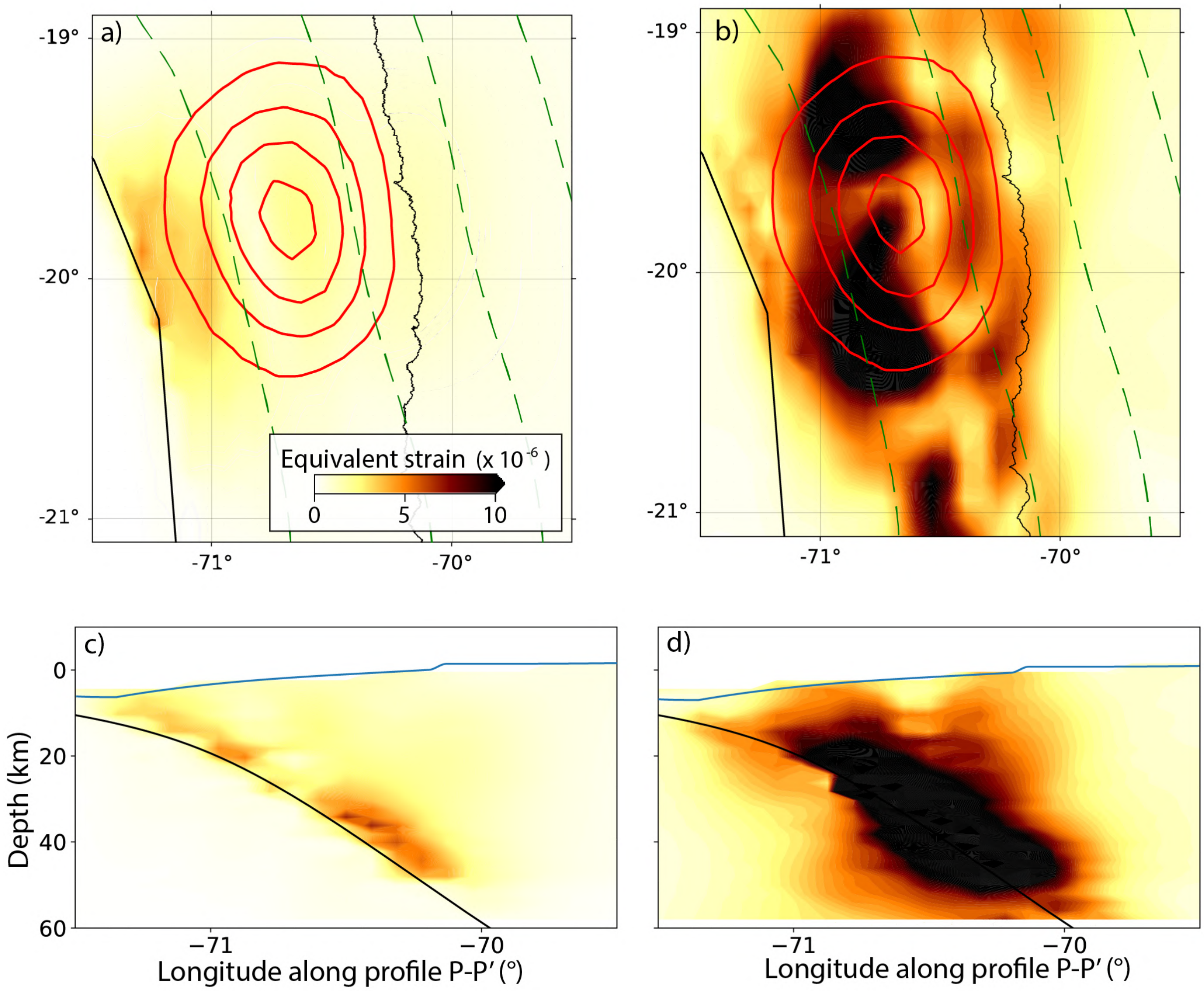

133 **Figure 19. Equivalent strain calculation**. Equivalent postseismic strain due to poroelasticity at a depth
134 of 12.5 km (a) and along a W-E cross section P–P' at 19.75°S. (c). Panels (b) and (d) show the
135 corresponding results for afterslip

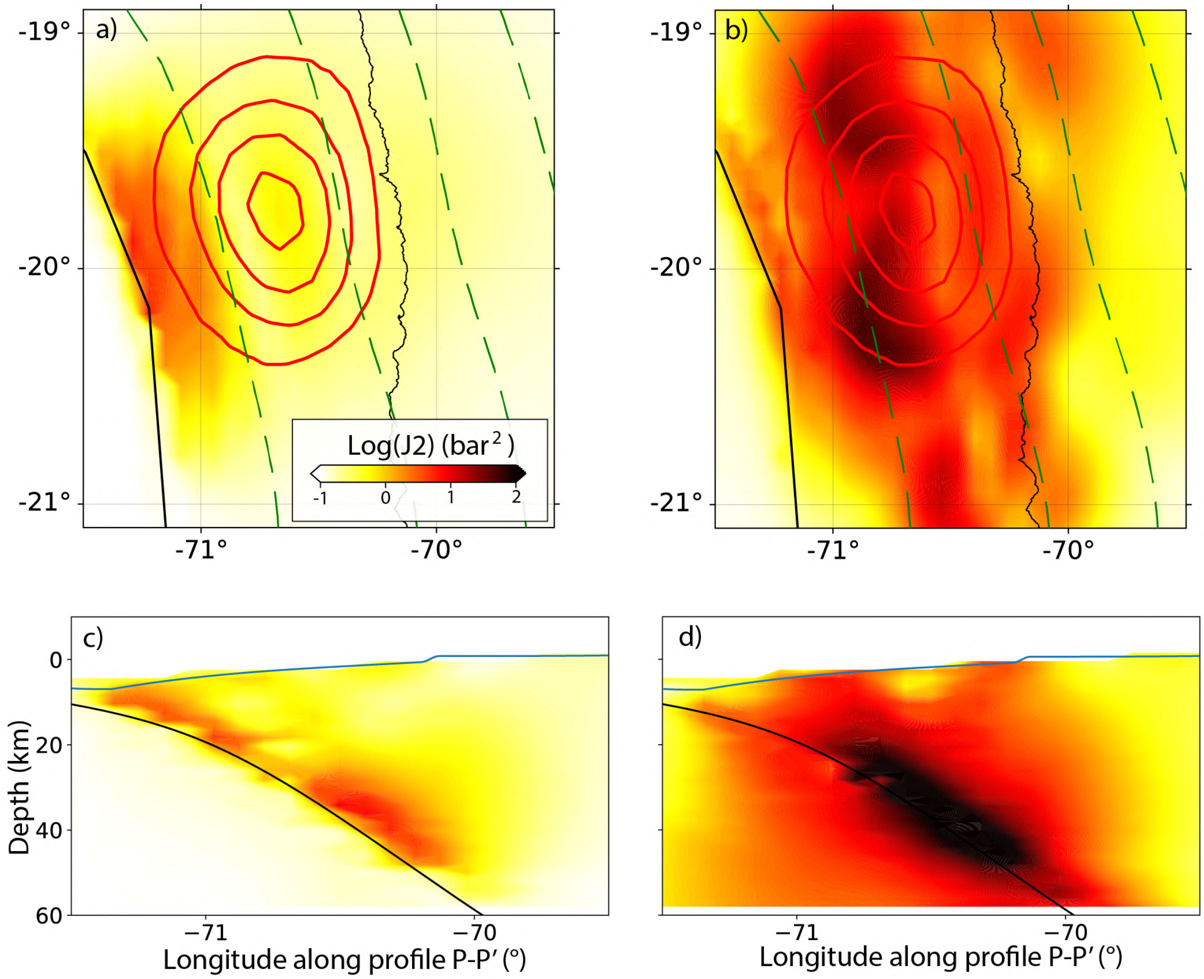

Figure 20. Second invariant of the deviatoric stress tensor calculation. As in Fig. 18, but for the postseismic second invariant of the deviatoric stress tensor, J_2

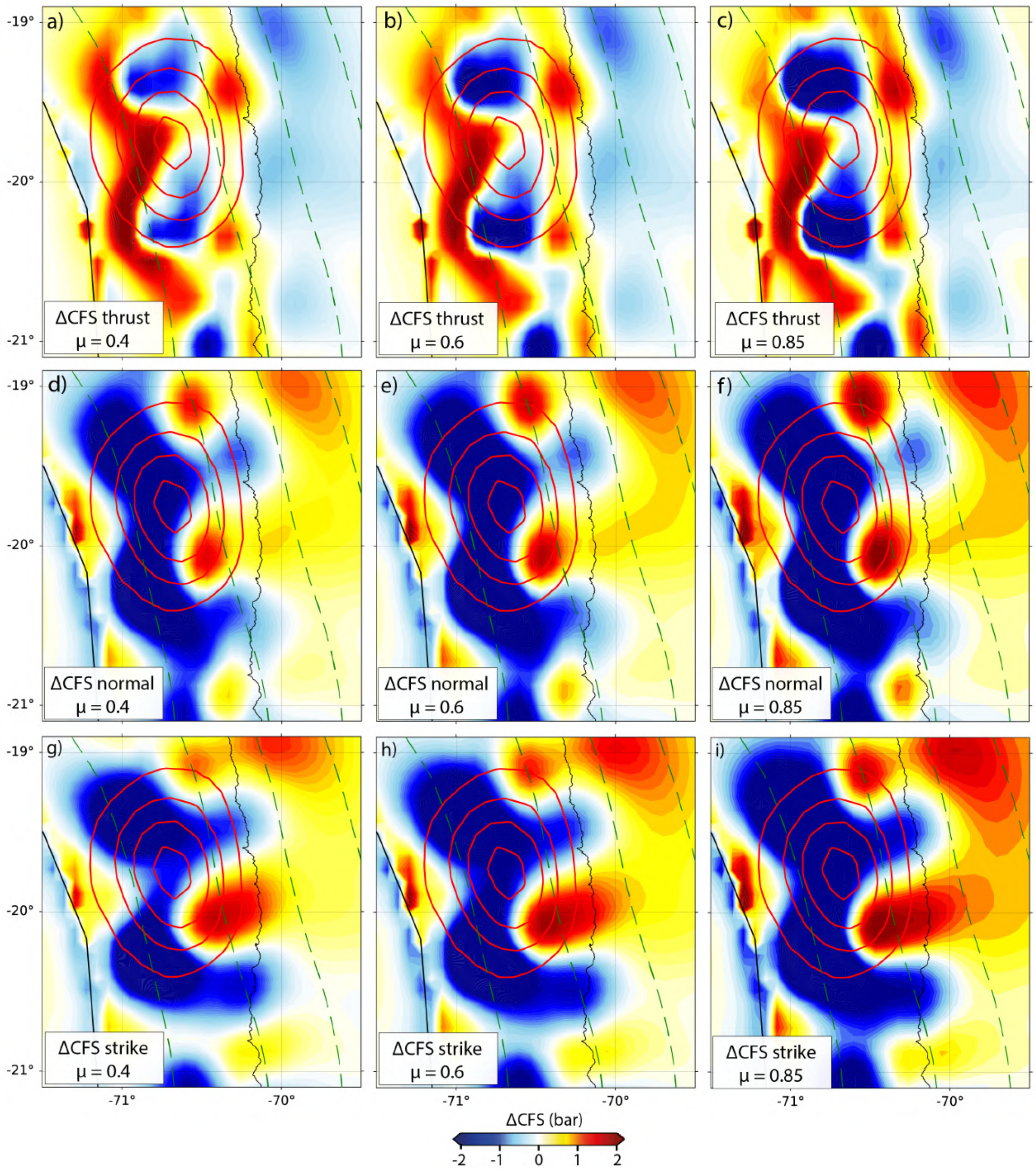

Figure 21. Impact of friction coefficient on Coulomb Failure Stress changes from afterslip at a cross-section at 12.5 km depth. Impact of the coefficient of friction μ on ΔCFS calculations due to afterslip. Panels (a–c), (d–f), and (g–i) correspond to thrust, normal, and strike-slip receiver faults, respectively. The geometry of the faults is shown in Fig. 3 of the main text.

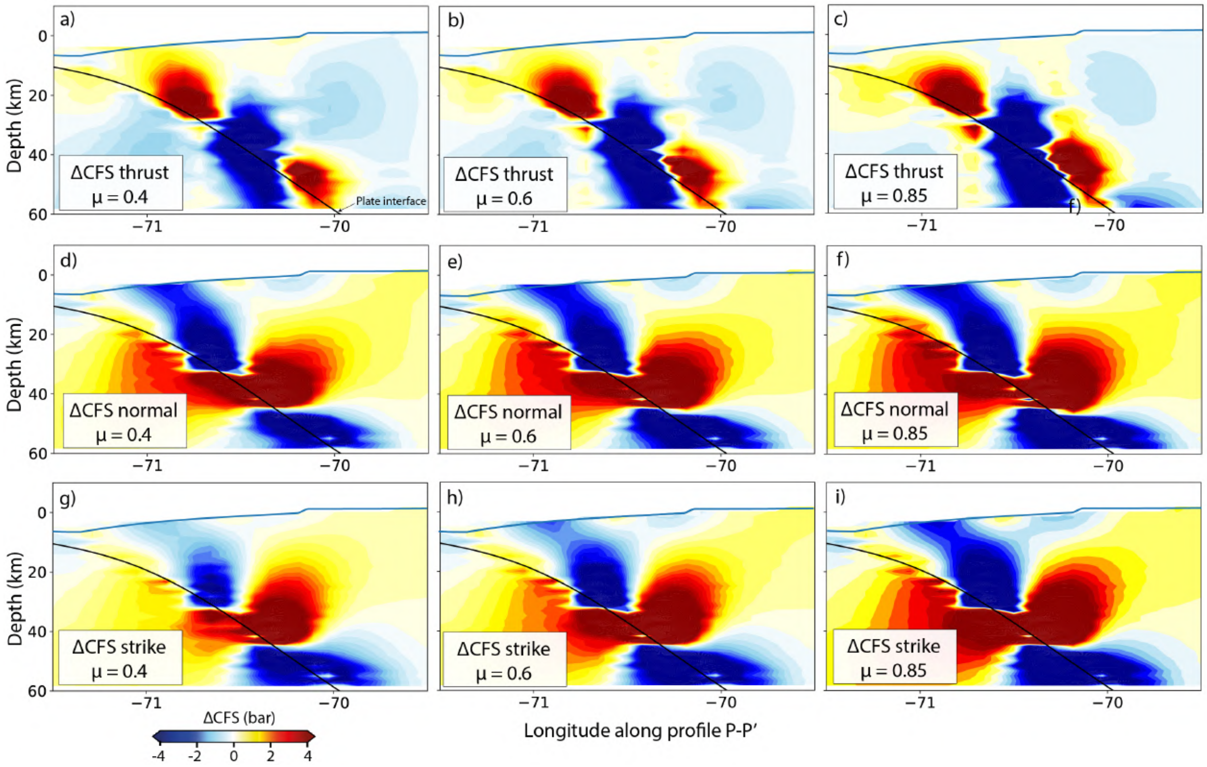

Figure 22. Impact of friction coefficient on Coulomb Failure Stress changes from afterslip along cross-section P–P'. As in Fig. 21 along section section P–P' at 19.75°S.

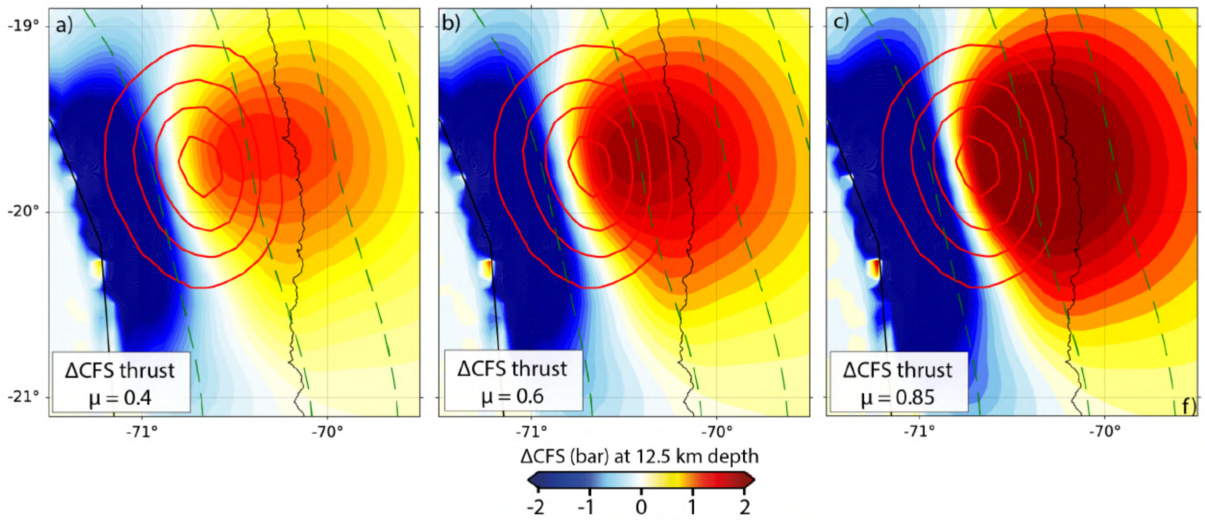

Figure 23. Impact of friction coefficient on Coulomb Failure Stress changes from poroelasticity at a cross-section at 12.5 km depth. As in Fig. 21, but for poroelasticity and only for a steep thrust receiver fault, given that the resulting ΔCFS is relatively insensitive to variations in fault geometry (see Figs. 3 and 4 in the main text). Similar effects are therefore expected for normal and strike-slip receiver faults.

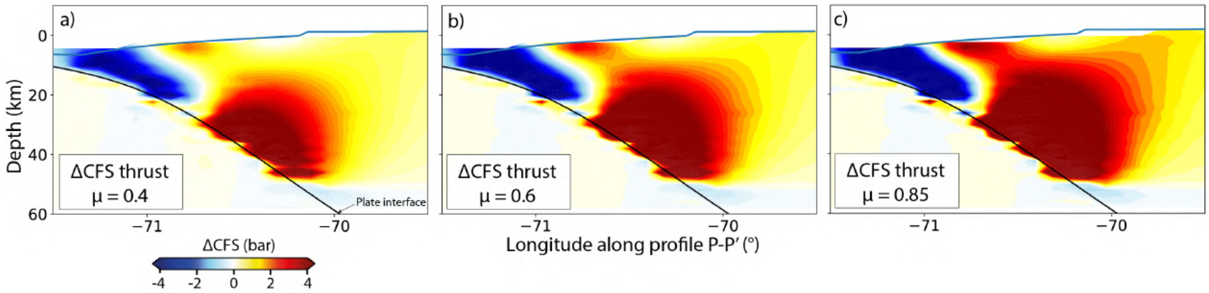

Figure 24. Impact of friction coefficient on Coulomb Failure Stress changes from poroelasticity along cross-section P–P'. As in Fig. 21 along a W–E cross section P–P' at 19.75°S.

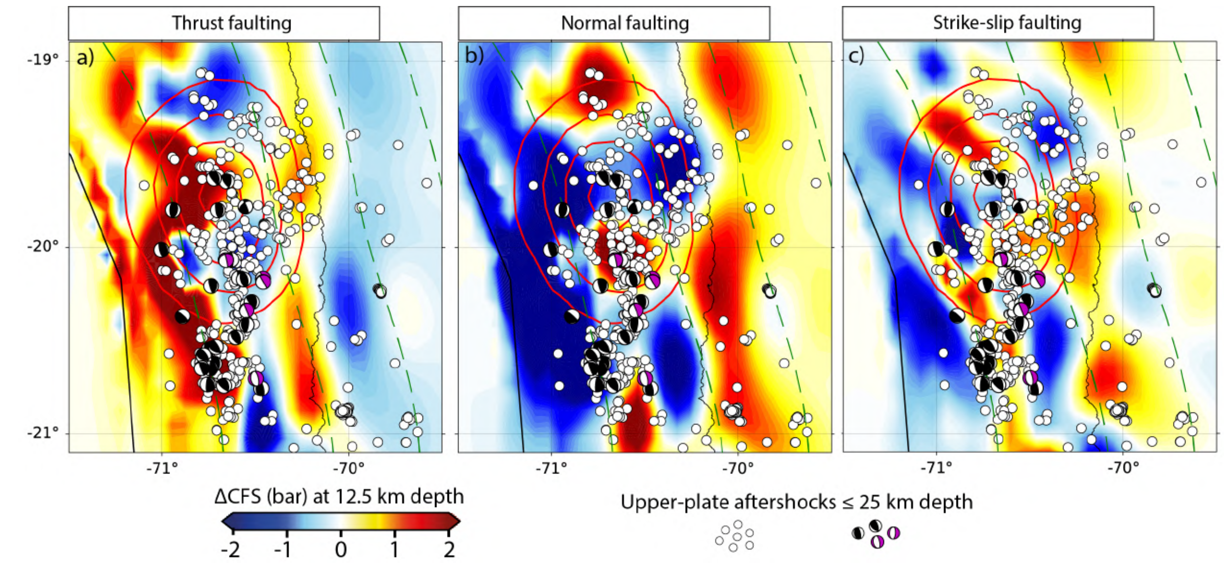

150 **Figure 25. Coulomb Failure Stress changes produced by afterslip considering an elastic-only model inversion along cross-section at 12.5 km depth**. Cumulative Coulomb Failure Stress changes (ΔCFS) after 270 days computer at 12.5 km depth due to the afterslip distributions resulting from an elastic-only model (Fig. 6a). Rake, dip, and strike angles are found in the main text, Fig. 3.

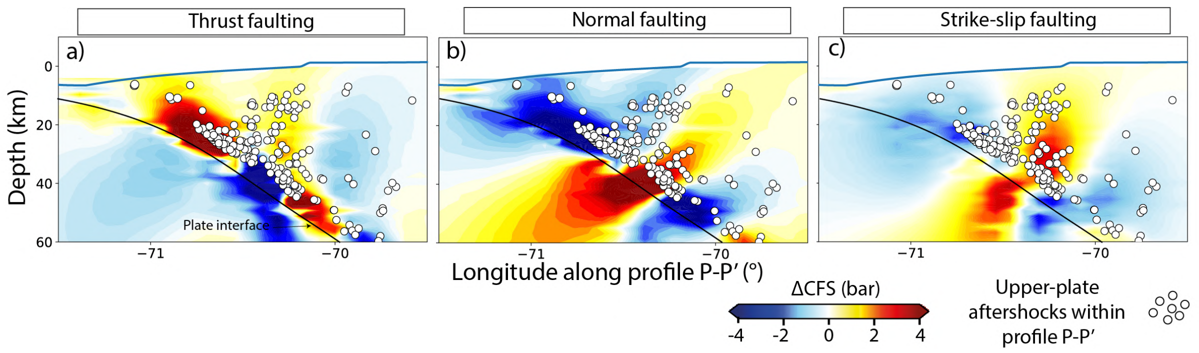

Figure 26. Coulomb Failure Stress changes produced by afterslip considering an elastic-only model inversion along cross section P–P'. As in Fig. 24 along a W–E cross section P–P' at 19.75°S.

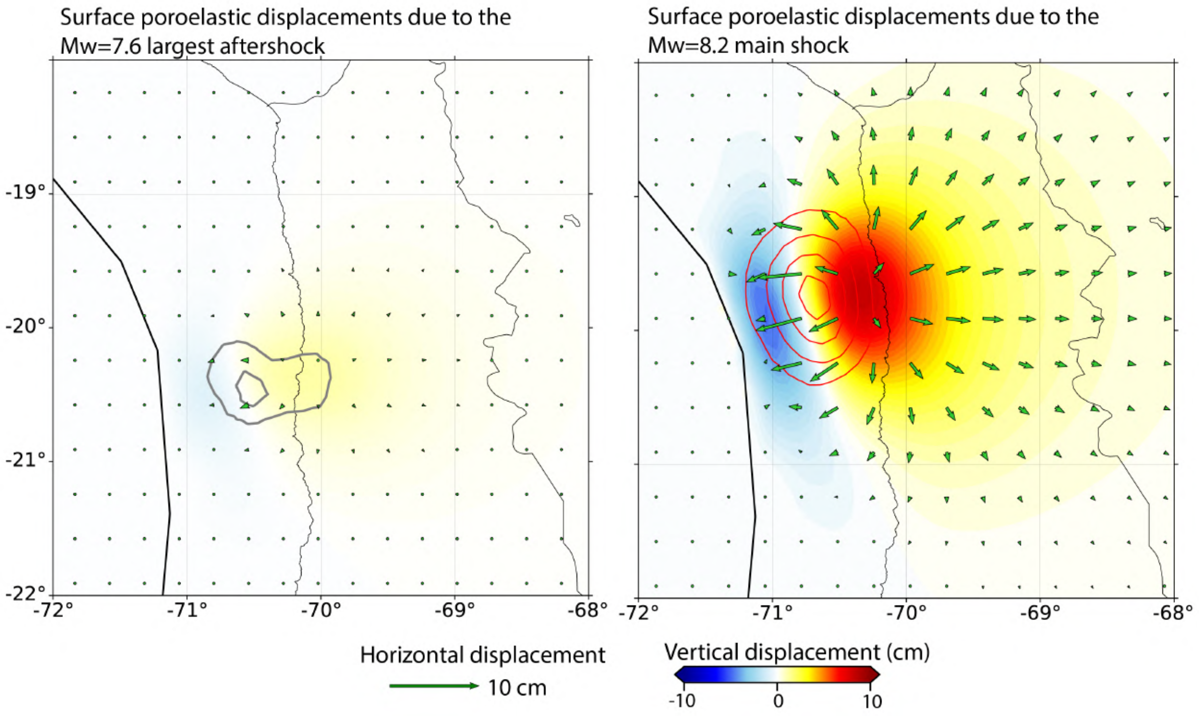

Figure 27. **Comparison of the surface displacements produced by the main shock and the largest aftershock M_w = 7.6**. Surface displacements produced by the largest aftershock (M_w 7.6) (a) and the main shock (b).

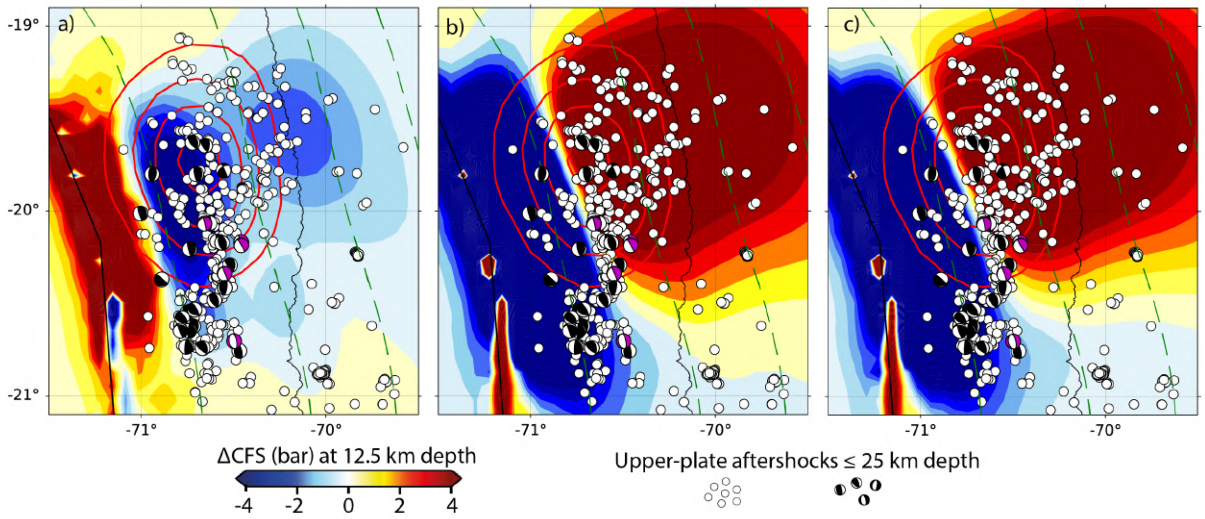

Figure 28. Coseismic Coulomb Failure Stress changes along a cross-section at 12.5 km depth. Coseismic ΔCFS computed at a depth of 12.5 km for thrust (a), normal (b), and strike-slip (c) receiver faults. The dip, rake, and strike angles are provided in the main text (Fig. 3).

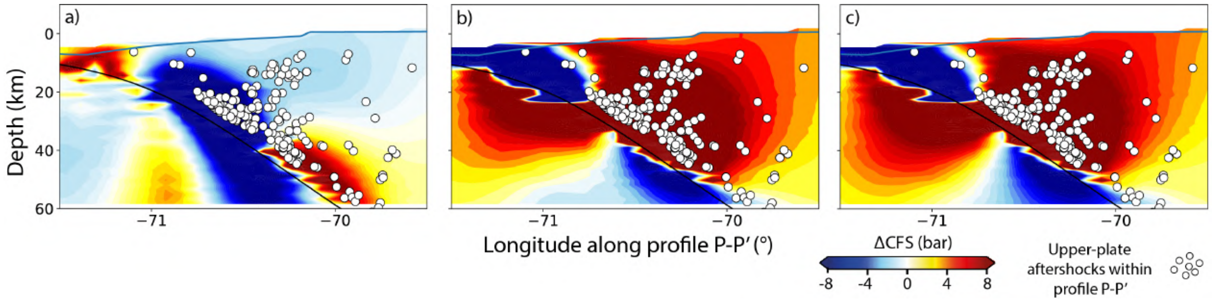

Figure 29. Coseismic Coulomb Failure Stress changes along a cross-section along cross section P–P'.As in Fig. 28 along a W–E cross section P–P' at 19.75°S.

References

1. Peña, C. *et al.* Role of Poroelasticity During the Early Postseismic Deformation of the 2010 Maule Megathrust Earthquake. *Geophys. Res. Lett.* **49**, e2022GL098144, DOI: https://doi.org/10.1029/2022GL098144 (2022).
2. Lin, Y.-n. N., Kositsky, A. P. & Avouac, J.-P. Pcaim joint inversion of insar and ground-based geodetic time series: Application to monitoring magmatic inflation beneath the long valley caldera. *Geophys. Res. Lett.* **37** (2010).
3. Shapiro, S., Patzig, R., Rothert, E. & Rindschwentner, J. Triggering of seismicity by pore-pressure perturbations: Permeability-related signatures of the phenomenon. *Pure Appl. Geophys.* 1051–1066, DOI: 0033-4553/03/061051-16 (2003).
4. Duputel, Z. *et al.* The iquique earthquake sequence of april 2014: Bayesian modeling accounting for prediction uncertainty. *Geophys. Res. Lett.* **42**, 7949–7957 (2015).
5. Okada, Y. Surface deformation due to shear and tensile faults in a half-space. *Bull. seismological society Am.* **75**, 1135–1154 (1985).
6. McCormack, K., Hesse, M. A., Dixon, T. & Malservisi, R. Modeling the contribution of poroelastic deformation to postseismic geodetic signals. *Geophys. Res. Lett.* **47**, e2020GL086945 (2020).
7. Peña, C. *et al.* Impact of power-law rheology on the viscoelastic relaxation pattern and afterslip distribution following the 2010 Mw 8.8 Maule earthquake. *Earth Planet. Sci. Lett.* **542**, 116292, DOI: https://doi.org/10.1016/j.epsl.2020.116292 (2020).
